# Artificial intelligence assisted preoperative planning and 3D-printing guiding frame for percutaneous screw reconstruction in periacetabular metastatic cancer patients

**DOI:** 10.3389/fbioe.2024.1404937

**Published:** 2024-07-29

**Authors:** Jichuan Wang, Zhiqing Zhao, Haijie Liang, Ranxin Zhang, Xingyu Liu, Jing Zhang, Swapnil Singh, Wei Guo, Taiqiang Yan, Bang H. Hoang, David S. Geller, Xiaodong Tang, Rui Yang

**Affiliations:** ^1^ Musculoskleletal Tumor Center, Beijing Key Laboratory for Musculoskeletal Tumors, Peking University People’s Hospital, Beijing, China; ^2^ Department of Orthopedics, Peking University First Hospital, Beijing, China; ^3^ Department of Orthopedic Surgery, Montefiore Medical Center, Albert Einstein College of Medicine, Bronx, NY, United States

**Keywords:** artificial intelligence, tripod technique, 3D printing, minimally invasive reconstruction, periacetabular metastasis

## Abstract

**Background:**

The percutaneous screw reconstruction technique, known as the “Tripod Technique,” has demonstrated favorable clinical outcomes in the management of metastatic periacetabular lesions, as evidenced by our prior investigations and corroborated by independent studies. Nevertheless, there is a steep learning curve in handling this technique, with possible complications such as intraarticular screw placement.

**Methods:**

Preoperative pelvic CT scans were acquired before surgery and utilized for the guiding frame design. A convolutional neural network model was trained with annotated data to identify the starting point and trajectory of each potential screw. A model boundary intersection detection technology was used to determine the optimal diameter and length of each screw. A non-rigid registration technology was matched with a prefabricated model of the body surface to design personalized anchoring skin pads. Finally, a polylactic acid-based guiding frame for intraoperative was custom-made with a 3D printer.

**Results:**

12 patients underwent a guiding frame-assisted Tripod procedure for treatment of periacetabular metastatic lesions. An intraoperative CT scan was performed in all cases to confirm screw trajectories. Among 36 screws that were implanted, 26 screws were implanted as designed. The remaining ten screws drifted, but all remained within the intra-osseous conduit without any complications. The mean surgical time was 1.22 h with the guiding frame compared with 2.3 h without the guiding frame. Following the surgical procedure, a noteworthy enhancement in pain management, as evidenced by a reduction in scores on the visual analog scale (*p* < 0.01), and an improvement in functional status, as assessed through the Eastern Cooperative Oncology Group score (*p* < 0.01), were observed when compared to the patient’s pre-operative condition.

**Conclusion:**

This proof-of-concept investigation demonstrates that the amalgamation of AI-assisted surgical planning and additive manufacturing can improve surgical accuracy and shorten surgical duration. While access to this technology is currently constrained during its early stages of development, it is anticipated that these limitations will diminish as the potential of AI and additive manufacturing in facilitating complex orthopedic procedures becomes more evident, leading to a surge in interest and adoption of this approach.

## Introduction

Metastatic disease often involves the periacetabular region, leading to pain, fracture, and mechanical instability (Issack et al., 2013). Symptomatic patients usually require a surgical procedure to improve mobility, quality of life, and to minimize the use of medication. In the 1980s, Harrington initially introduced a classification system for periacetabular metastasis and used modified total hip reconstruction to augment bone loss using Steinmann pins inserted into the iliac crest and embedded within the cement (Harrington, 1981). Over the years, various methods for positioning pins have been described, all of which aimed to improve hip or pelvic stability (Tillman et al., 2008; Lozano-Calderon et al., 2016). These open surgical procedures generally require extensive dissection, extended surgical duration, and considerable blood loss. They are associated with a high rate of postoperative complications, including infection, implant loosening, and hip dislocation, which all have been reported in up to 30% of patients (Ho et al., 2010; Lozano-Calderon et al., 2016; Rowell et al., 2019), which frequently results in the undesirable disruption of adjuvant care. Minimally invasive or percutaneous fixation generally avoids these complications, is well tolerated, provides immense pain relief, and permits for almost uninterrupted adjuvant treatment.

We first reported the percutaneous tripod reconstruction technique for the minimally invasive treatment of acetabular metastases in 2020 (Yang et al., 2020) and subsequently extended its application to non-periacetabular pelvic lesions (Yang et al., 2022). We have demonstrated that the tripod technique application is safe and well tolerated and that it meaningfully improves pain and functionality (Yang et al., 2020; Yang et al., 2021; Yang et al., 2022). It can be used as an independent procedure or in combination with other techniques, such as total hip replacement (Ibe et al., 2023). As a minimally invasive procedure, it allows for prompt or immediate adjuvant cancer treatment, which is an important consideration in cancer care.

Despite the simplicity of intraoperative set‐up and instrumentation of the Tripod technique, obtaining the requisite fluoroscopic views and troubleshooting intraoperative hurdles can be challenging, even for experienced orthopedic professionals. The determination of screw trajectory, length, and size represent real challenges, especially for less experienced surgeons. Artificial Intelligence (AI)-assisted radiographic analysis is an innovative emerging technology that may potentially revolutionize the field of orthopedic surgery. The merit of Machine learning (ML) algorithms resides in their ability to acquire knowledge from real-world applications and direct experiences, thereby augmenting their operational efficiency (Chen et al., 2022). Additive manufacturing and 3D printing have recently gained significant popularity across various fields, including surgery. Its advantages include customization, enabling the production of complex structures, increased efficiency, and the facilitation of biocompatible material creation. (Portnoy et al., 2023). The 3D printing process has been applied to models, implants, cutting guides, and other orthopedic surgery efforts, facilitating planning, increasing surgical accuracy, and reducing operation time, particularly in complex cases. ML has successfully enhanced various aspects of 3D printing in the medical field. Notable advancements include the integration of machine vision for multi-material 3D printing, improvements in 3D bioprinting processes, and robust quality control measures for 3D printed metal implants (Ding et al., 2023). These innovations highlight the transformative potential of ML in advancing additive manufacturing technologies. It is gradually becoming integral to the surgeon’s preoperative and intraoperative toolbox. However, few studies have integrated machine learning with 3D printing in orthopedic clinical scenarios to improve safety and efficiency. The current study aimed to investigate a novel integrative Tripod technique for treating acetabular metastasis using a 3D printing guiding frame with a machine learning algorithm.

## Materials and methods

### Patients

From October 2021 to March 2023, a total of 12 individuals diagnosed with metastatic cancer involving the periacetabular region of the pelvis were included. The indication for a surgical procedure was one or more symptomatic metastatic lesions involving the periacetabular area. In all cases, pelvic radiographs, MRI, and thin-layer computed tomography (CT) scans were obtained before the surgical procedure. Exclusion criteria included patients with protrusion or other evident disruption of the hip articular surface, as well as those patients with concomitant ipsilateral femoral head lesions. Two fellowship-trained orthopedic oncologists independently classified the acetabular defects using the Harrington and Metastatic Acetabular Classification (MAC) systems (Marco et al., 2000). Informed consent was obtained from all patients before the surgical procedure. Preoperative embolization was not performed in any of these cases. The diagnosis of metastatic bone disease was histologically confirmed by a bone pathologist either before or during the surgical procedure *via* a core needle biopsy sample. The study’s data collection involved chart review, which was conducted with the institutional review board’s approval. Data on patient characteristics, outcomes (such as survival, functionality, ECOG score), operative time, intraoperative bleeding, complications, and pain levels (measured using VAS) were either collected from the electronic medical record or at the time of clinic follow-up. Patients were asked to indicate their pain levels on a pain scale chart, with 0 representing the absence of pain and 10 representing the most severe pain, to assess VAS pain.

### AI-assisted guiding frame design

**FIGURE 1:**
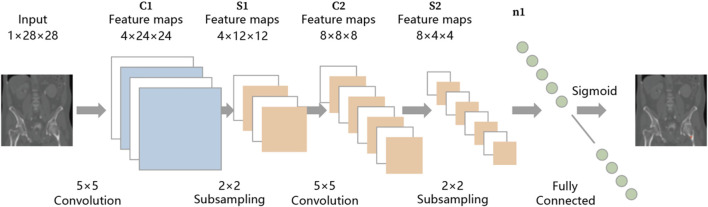
A schematic flow chart of the Deep neural network (DNN) model-based guiding frame design.

**FIGURE 2:**
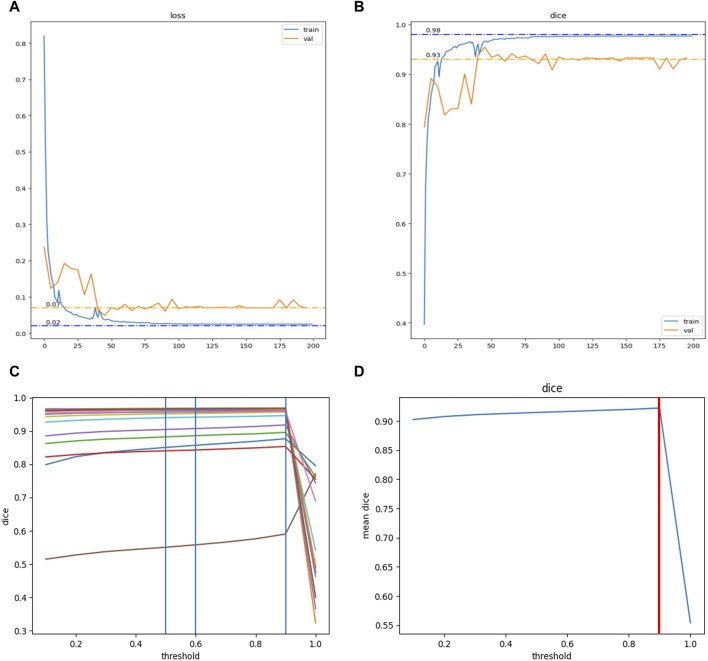
Training and hyperparameter tuning of the modified DNN model in this study. With 481 data sets, the model reached convergence after training for 200 epochs. **(A)** The Loss function converged to 0.02 in the training set (Blue curve) and to 0.07 in the validation set (Yellow curve). **(B)** The DiceCoefficient converged to 0.98 in the training set (Blue curve) and to 0.93 in the validation set (Yellow curve). Hyperparameter tuning for the model was further conducted. **(C)** The DiceCoefficient of each sample on different thresholds (range 0.0–1.0, step 0.1). **(D)** The average DiceCoefficient of all samples on different thresholds (range 0.0–1.0, step 0.1). The highest average DiceCoefficient reached the threshold of 0.9 (red line).

Two fellowship-trained professionals reviewed and labeled 481 annotated pelvis thin-layer CT scan data. Specifically, the trajectory, entry point, endpoint, and maximum allowable Tripod screw diameter were labeled in these cases. A Deep Neural Network (DNN) model was first trained using these data and thereafter modified to automatically recognize the optimal entry point and desired trajectory of each potential Tripod screw with input patient pelvis CT. Specifically, the axial CT data is segmented using three-dimensional multiplanar reconstruction (MPR) to generate a sagittal image set with 1 mm intervals. The neural network identifies the axial layer containing the entry and exit points within this sagittal image set. Subsequently, based on the identified axial layer information, the corresponding original axial images are retrieved. The neural network is then reapplied to these images to accurately determine the exact locations of the entry and exit points (Figure 1). Next, a self-designed model boundary intersection detection algorithm (Auto-BID) was used to determine the optimal diameter and length of each screw. Specifically, the two models (cortical bone and screw) intersect were determined by Voronoi Diagram-based triangulation. Furthermore, a self-designed skin surface recognition algorithm was used to determine and design personalized anchoring skin pads for the Tripod frame. The constructed model of anchoring pads was aligned with computed tomography (CT) data utilizing a dual-modality approach. This involved the application of an Iterative Closest Point (ICP) algorithm and a multi-modal registration technique, which facilitated affine transformation-based non-rigid model transformation (Figure 2). The Python programming language was employed to develop and implement these computational processes, operating within the Linux system environment.

### 3D printing of the guiding frame

After its design, the guiding frame was separated into three parts each of which were printed independently to accommodate the printer’s capacity. The stereolithography file of the guiding frame was imported into the 3D printer (FORMIGA P110, EOS GmbH, Germany) for polylactic acid-based frame printing. Subsequent to its fabrication, the frame underwent a visual inspection for quality assurance and was then precisely aligned in accordance with the specifications outlined in the software design. High temperature and pressure sterilization were applied before surgical use. The average production time from computer design to clinical application was about two to three workdays (Figure 2).

### Surgical technique

We utilized the previously described surgical technique, with the addition of the guiding 3D printed frame (Yang et al., 2020; Yang et al., 2021). The patient was placed on a transparent table supine and positioned with a small bump below the sacrum to elevate the pelvis off the table slightly. The guiding frame was assembled and positioned over the patient as pre-operatively planned. Bony landmarks were located using palpation and confirmed by assessing the fit of the frame to the patient’s anatomic contours. Multiple 2.0 mm guidewires were used to anchor and secure the frame, using predetermined guide holes that matched adjacent bony landmarks (pubic symphysis, anterior superior iliac spine, iliac wing, etc.). The position of the frame was confirmed using fluoroscopy. For each of the Tripod screws (anterior column screw, posterior column screw, and trans-columnar screw), 2.0 mm guidewires were firstly inserted through the guide hole in the frame in a stepwise manner., The obturator and iliac oblique views were primarily used for assessing the posterior column screw, the inlet, outlet, and obturator oblique views were used for assessing the anterior column screw, and the obturator oblique inlet and iliac oblique views were used for assessing the trans-columnar screw. At any point, a given guidewire can be replaced with a core needle for the purpose of biopsy. To further confirm the accuracy of the guiding frame, intraoperative CT scans were used for reconfirming the location of the guidewires. Once confirmed, the frame was disassembled, leaving the guidewires in place. A 5.5, 6.5, or 8.0 mm fully threaded cannulated screw (Zimmer Biomet) was selected and implanted through a small skin incision as previously described. The length of the screw was based on the preoperative AI-assisted determination and confirmed using fluoroscopy. Polymethylmethacrylate (PMMA) bone cement was used in any case involving osteolytic lesions within a bony cavity, especially those involving the acetabular dome, in which case the guidewire was replaced with a core needle for cement injection and thereafter switched back to the guidewire for screw fixation. Fluoroscopy was used for monitoring bone cement implantation to avoid extravasation (Figure 2). In the postoperative phase, patients were permitted to initiate weight-bearing on the surgically treated side as tolerated, commencing a few hours post-surgery, subject to individual patient comfort and readiness.

### Statistical analysis

GraphPad Prism 8.3.0 software (GraphPad Software, United States) was used for statistical analysis. Data were shown as mean—standard deviation. Student’s *t*-test was applied to the parameter changes between preoperative and postoperative data, and a *p*-value less than 0.05 was considered statistically significant.

## Results

**FIGURE 3:**
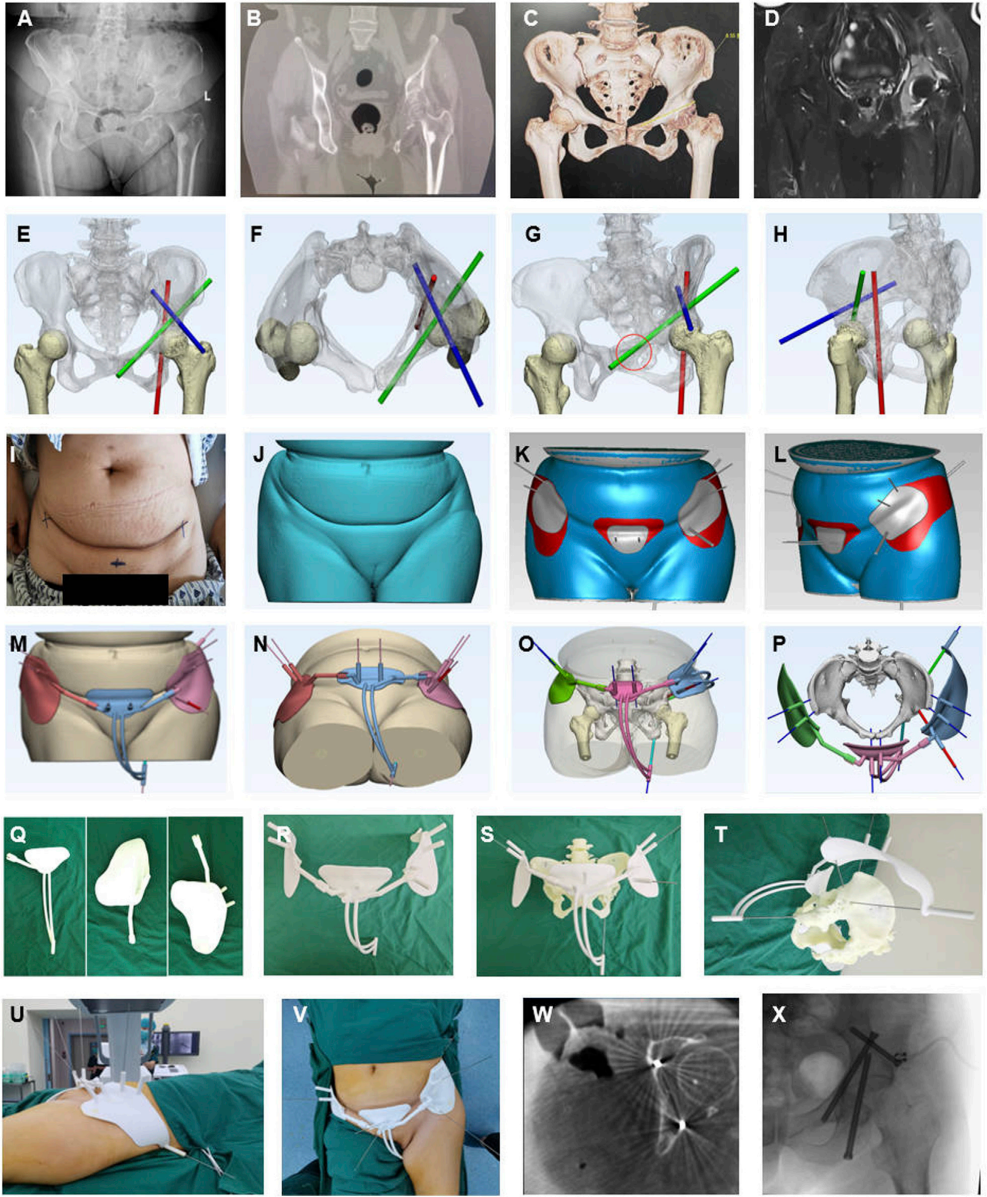
Representative case of AI-assisted preoperative planning and 3D-printing guide frame. **(A–D)** Preoperative X-ray, CT, CT reconstructed, and MRI radiographic examination showed a periacetabular lesion on the left. **(E–H)** The convolutional neural network algorithm is based on starting point and screw trajectory determination, Green for the antiror screw, Red for the posterior screw, and Blue for the trans-coloumlar screw. **(I–L)** The skin surface recognition algorithm was used for determining and designing personalized anchoring skin pads for the Tripod frame (Gery and red region on the skin. **(M–P)** Guiding frame generation with guiding tubes mimicking the screw trajectory, anchoring pads, and anchoring feet. **(Q–T)** 3D printing manufacturing of parts of the guiding frame, assembling and mimicking screw trajectory on the 3D printing pelvis model customed based on patient CT data with guide wires. **(U–X)** Intraoperative application of the guide frame, interoperative CT, and fluoroscopy validation.

The modified DNN model underwent a systematic process of training, accompanied by the tuning of hyperparameters. Utilizing a dataset comprising 481 labeled pelvic CT scans, the DNN model reached convergence after undergoing 200 training epochs. Upon evaluation, the model’s loss function converged to 0.02 in the training set and 0.07 in the validation set. Concurrently, the Dice Coefficient, a measure of model accuracy, converged to 0.98 in the training set and 0.93 in the validation set (Figure 3). Subsequent efforts were directed toward further refinement of the model’s hyperparameters. This process yielded a comprehensive analysis of the Dice Coefficient across various thresholds for each sample. The model exhibited optimal performance with the highest average Dice Coefficient at a threshold value of 0.9, a parameter subsequently adopted for future applications (Figure 3).

The demographic and clinical characteristics of the 12 patients in this study are provided in Table 1. There were seven women and five men with a mean age of 63.3 years (range 42–74 years). The preoperative American Society of Anesthesiologists (ASA) score was II in 2 cases, III in 8 cases, and IV in 2 cases. The ECOG evaluation was three in seven patients and four in the rest of five patients. The mean preoperative VAS pain score was 7.75 (range six–9). Two patients were bedbound, four were wheelchair-bound, and six could not ambulate themselves and required significant assistance (double crutch). All the patients had a known primary cancer and presented with a suspected metastatic pelvic lesion. In 9 patients, a core needle biopsy of the pelvis was performed during the procedure to both confirm the diagnosis histologically and to guide subsequent therapy. The most common primary diagnoses were breast cancer (n = 3), lung cancer (n = 3), renal cancer (n = 3), multiple myeloma (n = 2) and liver cancer (n = 1) (Table 1). 11 patients had a pathologic pelvic fracture or a cortical defect, identified on either plain radiographs or on CT scans. Radiographically, all patients presented with lytic lesions, while two patients also had evidence of radiodense matrix within the lesion. There were 11 patients with Harrington class-III lesions and one with Harrington class-II lesions. There were two patients with MAC classification Type 3b lesions (single-column deficiency with a dome and medial wall deficiency) and 10 patients with Type 4b lesions (double-column deficiency with a dome and medial wall deficiency).

**TABLE 1:** Patient demographics and baseline characteristics.

Case	Age (yr)	Gender	Diagnosis	MAC	Harrington classification	ASA	Fracture or complete cortical defect
1	55	F	Breast Cancer	4	III	II	Yes
2	42	F	Lung Cancer	4	III	III	Yes
3	52	F	Renal clear cell carcinoma	4	III	II	Yes
4	68	M	Multiple myeloma	4	III	III	Yes
5	74	M	Lung Cancer	4	III	III	Yes
6	68	M	Liver Cancer	4	III	IV	Yes
7	71	F	Breast Cancer	4	III	III	Yes
8	71	F	Renal Cancer	4	III	III	Yes
9	60	M	Renal cell carcinoma	3	III	II	No
10	59	F	Breast Cancer	4	II	III	Yes
11	71	M	Multiple myeloma	4	III	III	Yes
12	68	F	Lung Cancer	3	III	III	Yes

F, female; M, male; MAC, metastatic acetabular classification; ASA, American Society of Anesthesiologists (ASA) score.

The mean surgical time was 73.3 min (range, 45–110 min) (Table 2). Intraoperative bleeding was universally assessed as minimal, and no blood transfusions were needed. All patients were encouraged to get out of bed on the first postoperative day and ambulate with a walker under the supervision of a subspecialty-trained nurse. There were no surgical wound infections or healing problems. 11 patients received radiation therapy and postoperative systemic treatment, which included hormonal therapy, targeted therapy, immunotherapy, or cytotoxic chemotherapy, starting 1 week to 2 months after the surgical procedure. Six patients had modified plans of systemic treatment based on the pathological and biomolecular results gathered from the surgery.

**TABLE 2:** Clinical outcome of patients in this cohort.

Cases	Operative time (mins)	Follow-up (mo)	Screw implantation accuracy		ECOG		VAS score		Mobilization	
			As planned	Within the bone	Preop	Postop	Preop	Postop	Preop	Postop
1	110	13 (Alive)	2/3	1/3	4	1	9	2	Bed-bound	Single cane
2	100	12(Alive)	0/3	3/3	3	2	9	3	Double crutch	Single cane
3	90	10(Alive)	1/3	2/3	3	2	8	3	Double crutch	Independently ambulatory
4	70	8(Alive)	2/3	1/3	4	2	9	2	Bed-bound	Independently ambulatory
5	60	7 (Dead)	3/3	0/3	4	NA	8	3	Wheelchair	Single cane
6	70	7(Alive)	1/3	2/3	3	2	7	2	Double crutch	Single cane
7	60	6(Alive)	3/3	0/3	4	2	8	3	Wheelchair	Independently ambulatory
8	70	5(Alive)	3/3	0/3	3	2	7	2	Double crutch	Single cane
9	80	6(Alive)	3/3	0/3	3	1	6	2	Wheelchair	Independently ambulatory
10	70	4(Alive)	3/3	0/3	3	2	7	3	Double crutch	Independently ambulatory
11	55	4(Alive)	2/3	1/3	4	2	8	1	Double crutch	Single cane
12	45	3(Alive)	3/3	0/3	3	1	7	2	Wheelchair	Single cane

ECOG, eastern cooperative oncology group score; VAS, visual analog scale.

**FIGURE 4:**
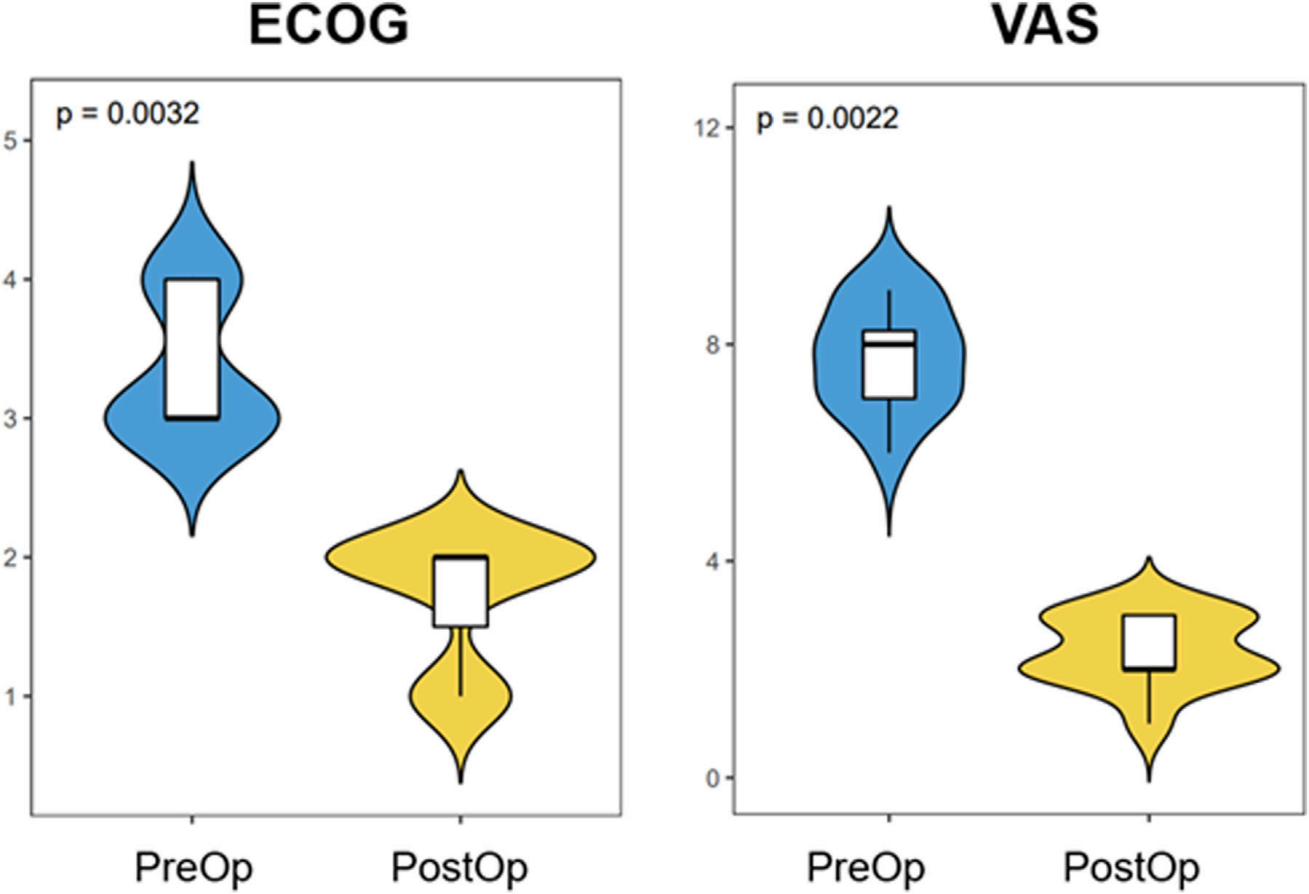
The comparison of VAS scores and ECOG scores between preoperative and postoperative.

One patient died 7 months after the surgery due to the progression of disease and complications from COVID-19. The mean follow-up time was 7.1 months (range, 3–13 months). Pain control improved by a mean of five levels on the VAS (*p* < 0.01) at 3 months postoperatively compared with preoperative pain control (Figure 4). At the most recent follow-up, 11 patients were alive. All patients achieved walking independently (5 patients) or with an assistive device (6 patients with a cane). Two patients subsequently regressed and again became wheelchair-bound due to either the progression of disease or spinal metastases. The mean postoperative ECOG score improved significantly (*p* < 0.01) compared with the preoperative score (Figure 4). Among the 11 patients who survived, five were engaged in chronic opiate use for pain management. Notably, only two of these individuals were utilizing opiates specifically for the management of periacetabular pain. These two patients also presented with disease progression around the affected periacetabular witnessed on a follow-up CT scan. At the most recent follow-up, all implants appeared unchanged, without evidence of loosening or failure. Screw trajectories were examined using intra or post-operative CT scans and compared with preoperatively planned trajectories. In total, 36 screws were placed collectively in 12 patients. Of these, 26 were placed exactly as planned. The remaining 10 screws were placed in a slightly altered trajectory, compared with the intended preoperative plan, however all of them remain within the bony conduit. There were no cases of intra-acetabular penetration, nerve injury or vascular insult. Four of these 10 screws could not achieve the pre-operatively planned length due to their altered trajectory and were replaced with shorter ones. All 4 shorter screws went through the osteolytic region caused by the tumor adequately spanned the region. Similar trends of improved ECOG scores were noticed in these patients, and no significant symptoms were reported postoperatively. The exact diameter of screws was achieved in all 36 screws as planned.

## Discussion

Thanks to advancements in both systemic and local adjuvant modalities, cancer patients are surviving for longer and living with their disease for increasing periods of time. While this progress is welcome, it results in even more patients developing symptomatic metastatic pelvic disease, an unintended consequence. Within the pelvis, the periacetabular region is one of the most common sites for tumor colonization and is frequently associated with pain and impaired ambulatory function. The complex anatomy of the pelvis coupled with often-times medically complicated patients presents many challenges and requires careful consideration. Non-operative treatment, such as radiation therapy, may prove inadequate for pain relief, particularly when pain stems from structural weakness. In such cases, surgical intervention offers value and warrants consideration.

For years, the primary approach to treatment has been open surgical management, which involves procedures like curettage and cementation, as well as total hip reconstruction using acetabular augmentation implants. These choices have offered pain relief and have enhanced function. However, they come at a cost, with inherent risks including substantial blood loss, extended hospital stays, prolonged recovery periods, peri-prosthetic infections, delayed wound healing, dislocation, higher treatment cost and surgical pain, requiring opioid pain medication. Approximately half of all patients undergoing these major open surgeries experience either a major or minor complication (Ho et al., 2010; Lozano-Calderon et al., 2016; Charles et al., 2017; Rowell et al., 2019). Many of these patients are older, have extensive whole-body tumor burden, and possess other comorbidities such as immunosuppression, impaired circulation, or suboptimal respiratory function, making them poor candidates for major surgery (Gao et al., 2020).

For these reasons, minimally invasive surgical techniques are becoming increasingly popular as an alternative to traditional open surgery. These techniques offer a stable reconstruction for ambulation and enable a quick resumption of systemic treatment while minimizing surgical complications (Table 3). Various methods have been outlined for the less invasive management of pelvic metastatic lesions (Ibe et al., 2023). As first reported by us, the Tripod technique is a percutaneous screw fixation for reconstructing acetabular structure (Yang et al., 2020). In addition, percutaneous-based cement augmentation, balloon Osteoplasty, ablation, and a combination of two or three techniques were reported (Ibe et al., 2023). Minimally invasive techniques reduce the likelihood of wound complications, deep infections, significant bleeding, transfusions, extended hospital stays, or the need for additional surgical procedures. Postoperatively, patients are allowed to bear weight as tolerated, avoid the risk of dislocation or implant-related complications, and are often discharged from the hospital in a very short time period. (Houdek et al., 2020; Lavignac et al., 2020; Plaud et al., 2022; Huang et al., 2023) (Table 3).

**TABLE 3:** The comparison of different surgical methods for acetabular metastatic carcinoma.

Year	Author	Surgical method	Surgical time (mins)	Intraoperative bleeding (mL)	Complications (e.g., infection, implant loosening, hip dislocation) (%)
2024	Current study	Guiding frame-assisted Tripod	73 ± 18	No blood transfusion	0
2020	Historical control Yang et al. (2020)	Tripod	137 ± 39	No blood transfusion	0
2020	Lavignac et al. (2020)	Total Hip Arthroplasty	-	-	30
2020	Houdek et al. (2020)	Harrington Reconstruction	318 ± 81	-	55
2022	Plaud et al. (2022)	Harrington Procedure	135 ± 29	1,433 ± 1,177	57
2023	Huang et al. (2023)	TAINVI robot + Tripod + Bone cement	45 ± 10	42 ± 8.37	0

To date, there has been a lack of agreement as to which minimally invasive method best enhances functional status or best alleviates symptoms such as pain (Hartung et al., 2016; Ibe et al., 2023). Selection often requires taking many factors into account, such as the location and dimensions of the metastatic lesion.

Despite its simplicity in terms of intraoperative set‐up and instrumentation, Tripod surgery can be technically demanding, particularly for inexperienced surgeons. Locating the optimal percutaneous entry point, obtaining the correct fluoroscopic views, and troubleshooting intraoperative hurdles can be challenging, especially in obese patients or in instances where anatomy is obscured or in cases where landmarks are hard to identify. A stepwise graphic guide for the tripod model has been previously reported, however translation from concept to practice can be challenging (Yang et al., 2021). A thorough understanding of anatomy and fluoroscopic views of the pelvis is essential.

Artificial intelligence, especially machine learning-based radiography analysis, has gained immense popularity in recent years and has the potential to revolutionize the field of orthopedic surgery (Oosterhoff and Doornberg, 2020; Chen et al., 2022). One of the foremost advantages associated with the application of machine learning in radiographic analysis lies in its capacity to distill information from annotated images obtained from real-world clinical settings, thereby enabling the incorporation of clinician expertise and consequently enhancing its evaluative proficiency. Although AI has been successfully applied in several orthopedic contexts, to date it has not been utilized or for preoperative planning in the context of minimally invasive surgery. Additive manufacturing and 3D printing have also gained significant popularity. Allowing for the creation of patient-specific models that improve the clinician’s visuospatial skills and enhance their understanding of anatomy (Oosterhoff and Doornberg, 2020; Chen et al., 2022).

In the current study, we innovatively combined a machine learning algorithm with 3D printing with the goal of further improving and streamlining percutaneous Tripod screw placement for the management of periacetabular metastatic bone disease. Our results demonstrate efficient and accurate surgical planning, shortened operation time and a facilitated surgical technique. The combination of AI and 3D printing also sparked optimism for advancing improved risk assessment tools to customize orthopedic care at every stage, from diagnosis to treatment. Preoperative radiography recognition under the vision of computers demonstrates encouraging outcomes in aiding decision-making, mitigating bias, handling large workloads effortlessly, and holding the promise of even surpassing doctors in specific tasks. Further research should focus on enhancing the workflow and refining the guiding frame. For instance, preoperative imaging could be transferred to a 3D printing center, allowing the guiding frame to be produced and shipped for surgical use. This approach is particularly beneficial for rural medical centers with limited equipment and professionals. The efficiency and accuracy of clinical ML-based 3D printing guiding frames require validation through further multi-center clinical trials.

We are still learning about various approaches to enhance and refine minimally invasive techniques for managing periacetabular osteolytic metastases (Rowell et al., 2019). Huang et al. reported surgical robot-assisted tripod percutaneous reconstruction (Huang et al., 2023). A passive surgical robot was applied, and Tripod screws were accurately inserted with shortened operation time (Table 3). Compared to the broader availability of 3D printing, prices of plastic-based guiding frames have become much more affordable than a costly surgical robot. Hence, institutional support and teams of engineers are also indispensable for the affordability, accessibility, and availability of surgical robots, especially for small/medium-sized hospitals. Additionally, surgeons also faced a long learning curve in robotic-assisted surgery to reduce actual intraoperative radiation and improve accuracy.

Preoperative planning for periacetabular metastasis requires careful consideration of procedure-type indications and evaluating comparable outcomes. Although clinical practice has demonstrated the benefit of the Tripod technique, biomechanical analysis to optimize the best configuration of screws fixation in different location and size of the osteolytic lesion still need further validation (Yang et al., 2020; Yang et al., 2022). Further, clinical observational studies or trials comparing different surgical techniques could answer questions regarding patient selection for proper procedures. Additionally, a more comprehensive outcome assessment system, one example is combining symptoms and ambulatory function, mainly designed for periacetabular metastatic patients, will facilitate outcome comparison among different techniques (Du et al., 2021).

There are several limitations in this study. Firstly, this study may present possible selection bias since we include patients with various cancer diagnoses, tumor sizes, and locations in a relatively small sample size. Patients included in this study were not randomly allocated to treatment. Although each patient’s treatment plan was made by multidiscipline consultation, the surgical team’s surgical treatment decisions were not based on standardized protocols. Although the guiding frame provides high accuracy in introducing all screws within the bone, some screws were not placed as planned. Highlighting further work on frame modifications for closer fit to body skin and firmly anchored during the surgery. Interestingly, we found that drift in one screw guide wire may transduce and affect the other two screws since the frame is rigid. We appreciate the frame design of assemblies and upgrades, including the flexibilities of the guiding tubes on the frame, which allow fine-tuning intraoperatively. Additionally, non-rigid materials such as silicone gel pads can be applied at the junction between the skin and the rigid frame, enhancing the stability and accuracy of frame installation. These will be included in our next version. Further, the current turnover of patient recognition to guiding frame ready for operation use still takes a few days, which may be too late for emergency patients with pathological acetabular fractures who suffer severe pain and are non-ambulatory. Additionally, we did not see a significant decrease in intraoperative fluoroscopy duration since we applied CT scan for screw position validation. The result of no intraarticular and extra-osseous penetration in this cohort may suggest that CT validation was not required. Meanwhile, fluoroscopies used in conventional Tripod procedures may overused when adapted with a guiding frame and need further optimization.

## Conclusion

For patients with metastatic disease to the periacetabular region, the Tripod minimally invasive technique has been shown to improve pain and function while minimizing patient risk and treatment cost. This proof-of-concept study further demonstrates that a 3D printed guide, built using deep learning algorithms, can successfully realize accurate and reproducible surgical outcomes. With further refinement and improvement, this approach may become a valuable tool in the larger surgical armamentarium. It may improve results in several circumstances including when surgeons have less experience, when patient anatomy is harder to visualize, or when minimizing surgical or anesthetic time is critical to safety.

## Data Availability

The raw data supporting the conclusions of this article will be made available by the authors, without undue reservation.
